# Soft-tissue metastasis revealing a pancreatic adenocarcinoma: one case report and a review of literature

**Published:** 2012-02-22

**Authors:** Yousra Akasbi, Samia Arifi, Mohammed El Idrissi, Mohammed Shimi, Faouzi Boutayeb, Abdelmjid El Mrini, Omar El Mesbahi

**Affiliations:** 1 Medical Oncology unit, Hassan II University Hospital, Fez, Morocco; 2 Orthopedic surgery unit, Hassan II University Hospital, Fez, Morocco

**Keywords:** Soft tissue metastases, pancreatic adenocarcinoma, computer tomography scan, rare, Morocco

## Abstract

Soft tissue metastases from pancreatic adenocarcinoma are rare lesions and can be the source of diagnostic confusion both clinically and pathologically. To our knowledge, one patient has been reported on with soft tissue lesions that ultimately disclose a pancreatic adenocarcinoma. We report here on a patient who presented with a metastatic soft tissue lesion in the trochanter, and the buttocks, as the initial manifestation of pancreatic adenocarcinoma. Soft tissue metastasis from pancreatic carcinoma is a rare finding. Clinicians should be aware that metastatic soft tissue lesions could be the initial presenting sign for pancreatic cancer. Also, the immunohistochemical staining for CK 7 and 19 may be helpful for the diagnosis of metastatic pancreatic adenocarcinoma.

## Introduction

Soft tissue (ST) metastases from pancreatic adenocarcinoma are rare lesions and can be the source of diagnostic confusion both clinically and pathologically. The most frequent sites of metastasis for carcinoma of the pancreas are the lymph nodes, lung, liver, adrenal glands, kidney and bone [1].

To our knowledge, one patient has been reported on with soft tissue lesions that ultimately disclose a pancreatic adenocarcinoma [2]. We report here on a patient who presented with a metastatic soft tissue lesion in the trochanter, and the buttocks, as the initial manifestation of pancreatic cancer.

## Case report

Written informed consent was obtained from the patient for publication of this case report and accompanying images. A copy of the written consent is available for review by the Editor-in-Chief of this journal.

A 56-year-old woman, without a medical or surgical history developed since March 2008 two masses. One localized in the left trochanter, and the other in the right buttocks. They are bulky, firm in consistency, irregular and painful, measured approximately 12 cm in the largest diameter, infiltrated the skin, extended towards the anal area and the posterior aspect of the upper left thigh, associated with inflammatory signs, but no systemic symptoms.

**Figure 1:**
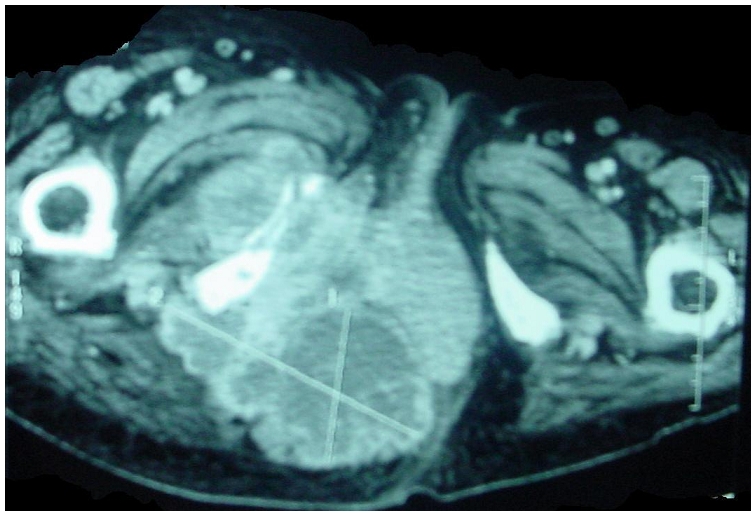
Computed tomography of the thigh: two tumors of the soft parts: left trochanter and retro ischiatic

One month later the patient benefited from a computed tomography scan of the thigh which showed two soft tissue tumors, one in the trochanter and the other in retro ischiatic (Figure 1). In May 2008, the resection of left thigh masse was done, the histopathological study documented a plasmocytoma, and immunohistochemical staining using monoclonal antibodies against CD138 was positive for CD138 and negative for CD20 and CD99. The patient was lost sight of the fact during three months, and then she was admitted in emergency for cholestatic jaundice.

Abdominal ultrasound and computed tomography scan showed a tumor at the head of the pancreas.

The second reading of the anatomopathologic specimen was consistent with soft tissue metastasis of pancreatic adenocarcinoma (CD138 was positive on the reactional Plasmocytes included in the tumor and not on the tumoral cells, CK7 and CK20 were negative). We concluded to a stage IV pancreatic cancer, and the patient was not operated.

According to ESMO guidelines, chemotherapy was started in September 2008 by gemcitabine at the dose 1000mg/m^3^ (on days 1,8 and 15 at 3-week intervals), after the first treatment , the clinical examination objectified a clear regression of the size and inflammatory signs of the buttocks’ tumor, the second treatment was envisaged but the patient died in October 2008 probably by acute severe anemia.

## Discussion

Carcinoma most commonly metastasises via the lymphatic system firstly to the regional lymph nodes and then into the general circulation. Dependent upon the primary site of tumour, metastases from carcinoma commonly occur to the lungs, liver and bone.

Distant metastasis to ST, defined as metastasis to skeletal muscle and subcutaneous tissues, are an uncommon presenting feature for primary solid tumours [3, 4] and represent less than 3% of soft-tissue malignancies [5]. Autopsy series have reported ST metastasis in 0.75%-9% of patients who died of metastatic carcinoma [5].

It is important to be able to distinguish the origin of a soft-tissue swelling as the management will depend significantly on the histological type. Although not in themselves infrequent, soft-tissue metastases are rarely encountered as a presenting sign of an occult cancer as by the time these appear, the patient has commonly developed symptoms pertaining to the primary tumour.

The most frequently reported locations for ST metastasis have been the back, chest wall, and abdomen [5].

The most commonly reported primary carcinomas to result in soft tissue metastases are those of the lung, kidney and colon [6], contrasting with those carcinomas which commonly metastasise to bone such as prostate, breast and thyroid which only very rarely metastasise to the soft tissues. Further, the most frequent histological diagnosis is adenocarcinoma [7].

When the muscular biopsy showed an “adenocarcinoma”, for men the primitive cancers were localized in the digestive tract (35%), kidney (20%), and lung (18%) and for women, the genital tract and breast (23.5%). When the muscular biopsy showed a “squamous-cell carcinoma”, the primitive cancers were localized in the lung (81%) for men and the cervix (64%) for women [8].

Use of a basic panel of immunohistochemical stains is recommended for defining the cell type and arriving at the correct diagnosis. Soft tissues metastasis has been reported as a common clinical presentation of occult malignancy and as an isolated metastasis in the patient with a known malignancy [5]. Only a small percentage of ST metastasis has been reported to occur in the presence of disseminated disease [5]. Distant metastases to the soft tissues are rare. There are few published case series, the majority of the literature containing only case reports [6]. These published case reports and small series provide limited perspective on which to base evaluation and management [9]. Damron and Heiner [3] who reported the largest series to date however had no cases where the patients’ primary site of carcinoma was of pancreatic origin.

In a series of 91 cases reported by Timothy et al, only one case described a pancreatic adenocarcinoma metastatic to soft tissue [2]. Mathis reported also 174 cases of soft tissue metastases [8]. Menard and Parache listed 247 cases in the literature since 1854 [10].

Recently, Jose Antonio Plaza reported One hundred and eighteen cases of soft tissue metastases. The primary tumor was located in the skin (19 patients), lung (13 patients), breast (13 patients), kidney (12 patients), colon and rectum (12 patients), uterus (8 patients), ovary (5 patients), head and neck (tongue, pharynx, larynx, nasal cavity, and mandible) (5 patients), esophagus (2 patients), stomach (2 patients), cervix (2 patients), small bowel (2 patients), bone (2 patients), adrenal gland (1 patient), eye (1 patient), testis (1 patient), urinary bladder (1 patient), and salivary gland (1 patient) and no primary tumor was located in pancreas [7].

The detection of soft tissues metastasis may affect staging and prognosis. Given the low-dose and non-contrast enhanced protocol, the computer scan (CT) helped localize the lesions and increase the diagnostic confidence as soft tissues metastatic lesions can appear hyperdense or hypodense as compared to the surrounding soft tissue.

Although magnetic resonance imaging (MRI) is not specific for soft tissue metastasis, it has been advocated as an indispensable tool for the diagnosis and treatment planning in patients with soft tissue malignancy [11].

However, a recent study showed that F-18 FDG PET/CT has higher sensitivity than MRI in detecting skin and ST metastasis [12]. This is supportive of the increasing role of F-18 FDG PET/CT in cancer patient management [13].

Although much rarer than primary soft tissue sarcomas, soft tissue metastases from carcinoma should remain a differential diagnosis in any patient presenting with a suspicious soft tissue metastases. Whilst the magnetic resonance scan appearances were suggestive of malignancy, they were not diagnostic of metastases.

Any painful soft tissue mass occurring in patients with unknown history of carcinoma, particularly with extensive peritumoral enhancement associated with central necrosis, is highly suspicious for skeletal muscle metastasis. A needle biopsy is necessary for proper diagnosis. It is very important to make a differential diagnosis between metastasis and primary cancer in these cases [14].

Treatment should be individualized according to the underlying disease and the prognosis.

The treatment for patients with pancreatic adenocarcinoma metastatic to soft tissue did not differ to a stage IV pancreatic cancer. Treatment of these patients with stage IV disease is often aimed at controlling symptoms and pain from the cancer. Chemotherapy is based on gemcitabine, while treatment with gemcitabine may be a reasonable choice, the use of a combination of gemcitabine and other cytotoxic agents, such as 5-FU, irinotecan, cisplatin and oxaliplatin, is not supported by an advantage in survival apart from capecitabine. However, this combination showed a survival advantage in a trial although it was not confirmed in another one. A meta-analysis of randomized trials with a combination of gemcitabine and platinum analogues seemed to suggest a role for this combination for young patients with good performance status. Nevertheless, the results of a large randomized trial comparing gemcitabine alone with gemcitabine plus cisplatin, presented at the last ASCO meeting, failed to show any benefit for the combination. Another therapeutic possibility is a combination of gemcitabine and erlotinib, recently approved by the FDA and EMEA on the basis of a randomized trial from the NCI of Canada. However, the very modest survival gain (2 weeks) and the high economic costs of the treatment question the role of this combination in metastatic pancreatic cancer. At the moment there is no evidence supporting the use of either cetuximab or bevacizumab in the overall setting of pancreatic cancer [15].

Certainly, the prognosis in the presence of ST metastasis should be considered, 77% of patients died of their disease within 1–22 months after ST metastasis was diagnosed [5]. This correlates with the reported median survival ranging from less than 5 months to no greater than 19 months after the diagnosis of ST metastasis [3].

## Conclusion

Soft tissue metastasis from pancreatic carcinoma is a rare finding. Clinicians should be aware that metastatic soft tissue lesions could be the initial presenting sign for pancreatic cancer. Also, the immunohistochemical staining for CK 7 and 19 may be helpful for the diagnosis of metastatic pancreatic adenocarcinoma.
